# Occupational therapy and primary care

**DOI:** 10.1017/S1463423618000452

**Published:** 2019-03-20

**Authors:** Marije Bolt, Tiska Ikking, Rosa Baaijen, Stephanie Saenger

**Affiliations:** 1 Occupational Therapist, Cordaan – Primary Care Team, Amsterdam, The Netherlands; 2 Occupational Therapist, Evean - Primary Care Team, Wormerveer, The Netherlands; 3 Occupational Therapist, Vermoeidheid&PijnCentrum - Lelystad, The Netherlands; 4 Occupational Therapist, Rolmaat - Abcoude, The Netherlands and COTEC president

**Keywords:** evidence-based practices, home based therapy, interventions, occupational therapy, primary care

## Abstract

This article is the first in a series of two articles about Occupational Therapy and Primary Care. This first article describes the health policy context in which primary health care should be strengthened. A definition of occupational therapy is given and the scope of the profession is explained. Based on a survey amongst the (experts of) member associations of COTEC, an overview is given of the main target groups and how occupational therapy is embedded and organized in different countries. In a position statement it is argued why occupational therapy can and should contribute to a comprehensive integrated primary care and challenges to strengthen the position of the profession are described.

## Purpose of the article

The purpose of this article is to outline the role of occupational therapy within primary care. This article informs consumers, academics, health service managers, professional associations and government bodies about occupational therapy in primary care, and informs service and policy development at a local, national and European level.

## Introduction: occupational therapy profession and primary care

Across Europe there are great differences in the availability of occupational therapy in primary care.

The need for integrated care that enables people to participate in decision-making and to self-manage their own health and well-being is widely recognized.

The challenges facing health care services include ageing populations and increasing numbers of clients with long-term conditions and multi-morbidities and all the social and economic consequences. These populations will benefit from an approach that is focused on possibilities and functioning rather than on a more medical treatment of symptoms (De Maeseneer and Boeckxstaens, 2012).

Occupational therapists can deliver an important contribution to the primary care workforce (Donnelly *et al*., 2014). A ‘clear fit’ has been identified between the holistic, health-promoting nature of occupational therapy and primary care (Donnelly *et al*., 2013). Occupational therapists recognize the importance of meaningful occupations in promoting mental, physical and social well-being. They are skilled in assessing the impact of developmental, physical and mental health conditions on a person’s ability to participate in activities that are important to them, and in devising intervention plans that facilitate occupational engagement (College of Occupational Therapists Ltd, 2015a).

Occupational therapists work in partnerships with other professions and can reduce the pressure on GP services. For example, in prevention and early intervention to prevent diseases or disability, reduce the impact of an illness and help support individuals in maintaining their healthy lifestyles (College of Occupational Therapists Ltd, 2015b). Within primary and early intervention services, occupational therapy interventions will reduce the risk of admission and re-admission into hospitals and other institutes from incidents such as falls (College of Occupational Therapists Ltd, 2016).

### Occupational therapy

According to Occupational Therapy Europe (OT-EU):

‘Occupational therapy is a profession concerned with improving well-being for persons of all ages through enabling occupations to promote health and participation in society. Occupational therapists do this by supporting persons’ engagement in occupations and activities that they want, need and choose to do in everyday life. Occupational therapists explore new ways of doing things by adapting activities and physical and social environments to improve function, capacity and participation. Occupational therapists work in partnership with those involved in the persons’ life, for example, family and carers, teachers and employers, to achieve persons’ and communities’ desired outcomes and promote an inclusive society’. (OT-EU Occupational Therapy Europe, n.d.)

For occupational therapy in primary care it should be added that occupational therapists work in the clients’ own environment where the activities take place at home, school work and/or social environment.

### Health policy context

In many countries, there is a shift from more institutional care to community care, both in mental and physical health care. The way health care is organized is not sustainable from both a financial and workforce point of view. In all countries in Europe, governments are struggling to reorganize the health systems and the workforce to meet the future needs. Although all health care systems aim to improve their population’s health, countries appear to organize their health care systems differently in response to their own political, economic, social, demographical and cultural context (Ros *et al*., 2000). In Western Europe emphasis on primary care is expected to be an answer to questions of rising costs and changing demand. Central and Eastern European countries are each in their own way struggling to fundamentally improve the performance of their entire health systems following their restoration of independence. Primary care, which used to be poorly developed in these countries, is now being developed to bring adequate and responsive health services closer to the population. Approaches and models of primary care reforms introduced have varied widely from country to country. Some countries have attempted systemic interventions combining legal, structural, organizational and financing reforms. Most countries however, touch on one or more aspects of primary care such as changes in the provision of services delivery by introducing evidence-based protocols; improving the generalist approach of primary care by improving the academic embeddedness of general practice; or introducing financial incentives for patients or providers to stimulate long-term relationships between single providers and patients (Kringos, 2012).

### Primary care

Primary care is at the top of the agenda of World Health Organization (WHO) and the European Commission. ‘It is undeniable that strong primary health care is foundational to achieving health for all, as well as today’s leading global health movements including Universal Health Coverage, Health System Strengthening, Health System Resilience, Integrated People-centred Health Services, and health related Sustainable Development Goals (SDGs)’ (World Health Organization, 2016). The Resolution WHA62.12 urging WHO member states to strengthen their health care systems through the values and principles of primary care. The WHO report 2008 articulates the need to bring responsive health services closer to the population and to provide people-centered care organized in primary care (World Health Organization, 2008).

In a recent report of the European Commission two of the five key conclusions are: A strong primary care guides patients through the health system and helps avoid wasteful spending and integrated care tackles a labyrinth of scattered health services to the benefit of the patient (European Union, 2017).

In the final consultation of the European Framework for Action on Integrated Health Service Delivery (WHO regional office for Europe) and Health 2020 it is stated that ‘Strengthening people-centred health systems… requires reorienting health care systems to give priority to disease prevention, foster continual quality improvement and integrate service delivery, ensure continuity of care, support self-care patients and relocate care as close to home as is safe and cost-effective’. Key elements in future health service delivery are investing in health through a life course approach and empowering people (World Health Organization, 2008). Strengthening the primary care level of health care systems have increasingly been considered to be of great importance to dealing with specific health care system challenges and improving the overall performance of a health care system (Kringos, 2012). Primary care is the first level of a health care system where people present their health problems and where the majority of the population’s curative and preventive health needs are satisfied (Starfield, 1994). Strong primary care is assumed to contribute positively to health system goals including (equity in) population health, sustainable health care expenditures and responsiveness of care (Kringos, 2012).

Not only the policymakers but also the patients in Europe are striving for accessible (primary) health care: ‘Sustainability strategies should include reducing the burden on secondary care by strengthening primary and community care, which is accountable for 80% of seeking treatment. Strong primary care systems are needed to provide continuous, comprehensive and coordinated care for the whole population’ (European Patient’s Forum, 2017).

An international comparative study to the strength of primary care resulted in listing the European countries with strong, medium and weak primary care (Kringos, 2012).

**Fig. 1:**
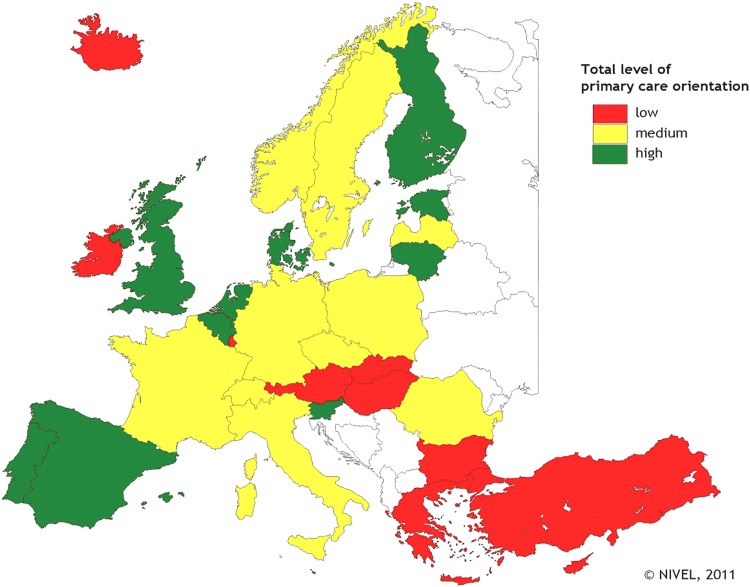
Countries with strong, medium and weak primary care, considering PC structure and key aspects of PC services delivery

The performance on all primary care structure dimensions (incl. primary care governance, economic conditions, workforce development) and services delivery process dimensions (incl. access, continuity, coordination, and comprehensiveness of primary care) were taken into consideration (Figure 1).

#### Primary care definitions

Primary care is the entry level of a health care system providing accessible, comprehensive care in an ambulatory setting to patients in their own context on a continuous basis. Primary care coordinates the care processes of patients across the health care system (Starfield, 1994). Primary care ideally provides accessible care to all patients with any kind of health problems regardless of age, sex or any other personal characteristic (World Health Organization on behalf of the European Observatory on Health Systems and Policies, 2006).

These characteristics are also mentioned in the definition of the WHO regional office for Europe. Primary health care is ‘health care received in the community, usually from family doctors, community nurses, staff in local clinics or other health professionals. It should be universally accessible to individuals and families by means acceptable to them, with their full participation and at a cost that the community and country can afford’ (World Health Organization, 2008).

Primary care exhibits features of person-centeredness, comprehensiveness, integration, continuity of care, participation of patients, families and communities. This requires health services that are organized with close-to-client multidisciplinary teams responsible for a defined population, collaborate with socials services and other sectors, and coordinate the contributions of specialists and community organizations (World Health Organization, 2016).

In July 2014 The Expert Panel on Effective Ways of Investing in Health (EXPH, 2014) adopted the Report *on Definition of a frame of reference in relation to primary care with a special emphasis on financing and referral systems.* In this report, the EXPH publishes a core definition of primary care in which occupational therapy is mentioned as one of the professions active in primary care teams.

‘*The Expert Panel considers that primary care is the provision of universally accessible, integrated person-centred, comprehensive health and community services provided by a team of professionals accountable for addressing a large majority of personal health needs. These services are delivered in a sustained partnership with patients and informal caregivers, in the context of family and community, and play a central role in the overall coordination and continuity of people’s care.*

*The professionals active in primary care teams include, among others, dentists, dieticians, general practitioner’s / family physicians, midwives, nurses, occupational therapists, optometrists, pharmacists, physiotherapists, psychologists and social workers*’. (EXPH, 2014).

### Occupational therapy and primary care

There are many differences in the European countries regarding the definition and criteria of primary care. This is the same for occupational therapy in primary care. For instance, in the UK primary care is defined as being ‘the first contact’ in health care. In the Netherlands the way the occupational therapy services are financed defines whether it is primary care. Other countries have the referral system as criteria. Referral systems are often directly connected with financing and are as such an important issue. Some countries have the environment/context where the occupational therapist works as criteria which can be home, school, workplace and or private practice or are called ‘*community based*’. The WHO definition states primary care services *… should be universally accessible to individuals and families by means acceptable to them…* which in fact would exclude all care that has to be paid for by the person.

Occupational therapy services in primary care are not only aimed at the individual in health care but also in groups and communities and are delivered in public health and the social area. Occupational therapy plays an important role in working with those who have chronic conditions etc., but also in working with the well. As the financing systems of the different areas – health insurance, social insurance, municipality – are separately organized and occupational therapy services are often delivered at the borders and both sides of these areas, the financing is a major challenge. As this article aims to give an overview of the state of the art of occupational therapy in primary care in European countries and to support those countries developing occupational therapy in primary care, all possible contexts are taken into account, even though in some countries they won’t meet all the criteria set by the definitions above.

#### Statement of the position taken by COTEC (Saenger, 2016)

Occupational therapists critically embrace the definition of the concept of Positive Health. ‘The ability to adapt and to self-manage, in the face of social, physical and emotional challenges’ (Huber *et al*., 2011) and find that health as ‘a state of complete physical, mental, and social well-being and not merely the absence of disease or infirmity’ as defined by the WHO in 1948 (World Health Organization, 2006) seems not applicable in these times with ageing populations and increasing numbers of patients with long-term conditions and multi-morbidities.

Occupational therapists possess professional skills that enable them to work with a wide range of clients of all ages who are faced with limitations in their participation due to physical, mental and/or social economic causes.

Occupational therapists recognize the importance of meaningful occupations in promoting mental, physical and social well-being. They are skilled in assessing the impact of developmental, physical and mental health and social conditions on a person’s ability to participate in activities that are important to them, and in devising intervention plans that facilitate occupational engagement.

Although occupational therapists have the medical knowledge, their focus is on functioning and participation. They operate in both health and social systems. Occupational therapists have distinct knowledge of the significant impact that daily habits and routines have on individuals’ health and well-being. They are experts when it comes to finding – in partnership with their clients – solutions for situations in which there is a gap between the clients physical and/or mental abilities and the skills that are necessary to perform daily activities. Occupational therapists support people to live safely at home, prevent unnecessary hospital or other institutional admissions and prevent an excess of care-use at home.

Because occupational therapists address the entire area of daily living, they are used to work with every other professional in both health- and social care and also with professionals in the more technical fields, such as architects, ICT, product developers and designers. This makes occupational therapists fully equipped to play a central role in interprofessional teams in primary care and integrated care.

Occupational therapy services should be available and accessible in primary care in all health- and social care systems across Europe. Having an occupational therapist in a multi-professional team could be helpful in identifying ways to integrate the health and social services to better effect. Looking for solutions that address the impact of illness or disability and how individuals participate in society is the key to robust integrated health care delivery systems.

## Survey

To make a start with the investigation to what extent members of COTEC differ in the strength of occupational therapy in primary care, the project group sent out surveys to all 30 members of COTEC.^1^ In total, 20 surveys were returned, two from the same country. Since each country in Europe is in a different stage of development of their primary care information infrastructure, inevitably, some countries have a more comprehensive, up-to-date or reliable set of data than other countries. These surveys were ordered and analyzed by eight themes: total number of occupational therapists in private practice, summary of the profession, organized interest group within National Association, payment system, accessibility, client groups, main working area and main challenges. The project group expected that countries vary in the strength of occupational therapy in primary care, which can be explained by variation in their political-economical, cultural and health care system contexts. Strong primary care is expected to be beneficial to important health care system outcomes (Kringos, 2012). Currently, countries with relatively strong PC have higher total health care expenditures than countries with relatively weak PC in Europe. The results confirm that strong PC has a positive impact on population health, reducing disparity in health, and avoiding unnecessary hospitalizations. Patient perceived quality of care is not related to the strength of PC (Kringos, 2012).

### Number of occupational therapists

In total, 11 member associations of COTEC have returned the survey with a positive answer on the question whether they have occupational therapists working in primary care. Their number varies between 6 (Luxembourg) and 1400 (Norway).

Out of the 30 member associations of COTEC, 26 completed the 2016 summary of the profession on the topic of ‘occupational therapists working in private/independent practice’ (COTEC Executive Committee, 2017). 23 member associations of COTEC have occupational therapists working in private/independent practice and their number varies between 3 (Iceland) and 26 000 (Germany).

We choose to compare the primary care and private/independent practice data in Table 1.

**Table 1:** Number of occupational therapists working in primary care or private/independent practice

COTEC member	Number of occupational therapists working in primary care according to 2016 survey	Number of occupational therapists working in private/independent practice according to 2016 summary of the profession
Austria	No answer (Na)	1200
Belgium	400	1057
Bulgaria	Na	0
Croatia	Na	20
Cyprus	Na	43
Czech Republic	Na	5
Denmark	400	821
Estonia	Na	Na
Finland	Na	300
France	800	800
Georgia	Na	0
Germany	Na	26000
Greece	Na	Na
Iceland	Na	3
Ireland	500	120
Italy	Na	1300
Latvia	Na	19
Lithuania	Na	Na
Luxembourg	6	6
Malta	Na	8
The Netherlands	740	900
Norway	1400	12
Portugal	68	Na
Russia	Na	0
Serbia	Na	50
Slovenia	Na	10
Spain	75	1560
Sweden	Na	850
Switzerland	1200	850
United Kingdom	700	752

Some differences in numbers are remarkable. That might be due to differences in interpretation of primary care and private/independent practice and/or the position and level of knowledge of the person who answered the questions.

### Special interest groups

As a special interest group is often started when a country wants to develop or has developed a special area of practice, in this case primary care, this item was included in the survey.

There are four interest/expert groups about occupational therapy in primary care identified according to the returned surveys. Both the countries with the largest and smallest number therapists working in private practices have established such a group.

### Funding of services

Out of 18 answers (with several payments systems existing next to each other), the most common payment system is health insurance, followed by clients paying for themselves, payment systems of the municipality and other (government, general taxation, public system and private providers) (Table 2).

**Table 2:** Most common payment systems for occupational therapy in primary care

COTEC member	Health insurance	Municipality	Self	Other
Austria	X		X	
Belgium	X	X	X	X
Bulgaria	No answer (Na)			
Croatia	Na			
Cyprus	Na			
Czech Republic	X			
Denmark	Na			
Estonia	Na			
Finland	Na			
France	X		X	
Georgia	Na			
Germany	Na			
Greece	Na			
Iceland	Na			
Ireland				X
Italy	Na			
Latvia	Na			
Lithuania	Na			
Luxembourg	X		X	
Malta	Na			
The Netherlands	X		X	X
Norway		X		
Portugal				X
Russia	Na			
Serbia			X	
Slovenia	X		X	
Spain	X	X	X	X
Sweden	Na			
Switzerland	X		X	
United Kingdom	X			X
Total	9	3	9	6

### Referral systems

Out of 14 countries, 11 offer the possibility for occupational therapy through direct access, two of them as being the only option to start occupational therapy treatment. Two other member associations outlined the fact that no referral or direct access check is needed when clients pay for the occupational therapy treatment themselves.

### Main working area

The main group of clients being treated by occupational therapists in primary care is the elderly. Three countries do not offer occupational therapy in primary care for this group at all. They do offer specific occupational therapy in primary care for adults (with mental disabilities) and children.

### Main challenges

There is no single source of information that provides basic information on the organization and delivery of primary care services across Europe. A major cause is the lack of a common definition of primary care that can capture the variation in organization and services delivery models (Kringos, 2012). The lack of comparable information on primary care across Europe, limits opportunities to provide benchmark information on the functioning of primary care to policymakers (eg, to measure the impact of healthcare policies on primary care), identify strong features or options to improve the functioning of primary care, and explain variation in the strength of primary care between countries (Kringos, 2012). The same applies for occupational therapy.

The results of the survey show that a clear and well-accepted definition of primary care is needed to be able to get the right data of occupational therapists working in this area. Further and more in depth research is needed to describe the financing systems of occupational therapy services across Europe.

The main challenge for occupational therapy in primary care, according to the analyses of the survey, is the difficult accessibility to occupational therapy services caused by:

- A lack of occupational therapists in primary care;
- A lack of knowledge among the general public and the medical professionals regarding the services of occupational therapy in primary care;
- A fragmentation of the organization of health care and social services;
- The complexity of the financing structures;
  The other challenge is the professional development and the limited possibilities to build a robust knowledge base.
- Most occupational therapist work solitary in primary care without the possibility to be supported and learn from each other;
- If occupational therapists work in a multidisciplinary team, they experience unrealistic caseloads and inadequate resources due to a high number of referrals (Tinelly and Byrne, 2016).
- Scope of the profession;

- Referrals are:
  - for very specific OT tasks which limits the scope of the profession;
  - or the scope is too broad which results in a lack of focus.

- Cooperation between practice, education and research are not optimal.
- Lack of research on (cost) effectiveness.

## Conclusion

Strong primary care will not emerge spontaneously: it will require continuous efforts to maintain, restore or strengthen its functions to deliver high-quality primary care (Kringos, 2012).

Occupational therapy has a lot to offer in primary care especially if it is embedded in the local, regional and national health and social systems.

Although occupational therapy is named as a key profession in the core definition of Primary Care (EXPH, 2014) (new reference has been added) and is (well) established in primary care at least 14 European countries, occupational therapy is according to Professor Jan de Maeseneer still ‘the most underused profession in primary care’ (De Maeseneer, 2017).

Reasons are the way primary health care is organized and financed which hinders the accessibility of occupational therapy in primary care, the relatively small number of occupational therapists and the unfamiliarity with the profession by policymakers, referrers, other professionals and the general public.

To develop and strengthen the position of the profession in primary care, promotion to policymakers and public is needed, as is evidence of the (cost) effectiveness of the interventions.

This article and the second article describe and capture just a phase of the development and state of the art of occupational therapy and primary care. Seeing the developments in health and social care and especially in primary care in Europe, and given the efforts and results of COTEC and OT-EU and its members in promoting the profession on European and national level, it is to be expected occupational therapy will follow and will be developed, expand and established in more countries and in many ways.

## Footnotes

1
The survey is available by mailing the author.
